# Costs of screening children for hearing disorders and delivery of hearing aids in China

**DOI:** 10.1186/1472-6963-9-64

**Published:** 2009-04-16

**Authors:** Rob Baltussen, Ju Li, Li Dong Wu, Xiao Hui Ge, Bai Yu Teng, Xi Bin Sun, Rui Han, Xiao Li Wang, Bradley McPherson

**Affiliations:** 1 Department of Public Health, Radboud University Nijmegen Medical Center, Nijmegen, the Netherlands; 2 China Rehabilitation Research Centre for Deaf Children, Beijing, PR China; 3 GuangXi Zhuangzu Autonomous Region Rehabilitation, Research Center for Deaf Children, Nanning, China; 4 Division of Speech and Hearing Sciences, Faculty of Education, University of Hong Kong, Hong Kong

## Abstract

**Background:**

The burden of disease of hearing disorders among children is high, but a large part goes undetected. School-based screening programs in combination with the delivery of hearing aids can alleviate this situation, but the costs of such programs are unknown.

**Aim:**

To evaluate the costs of a school-based screening program for hearing disorders, among approximately 216,000 school children, and the delivery of hearing aids to 206 children at three different care levels in China.

**Methods:**

In a prospective study design, screening and hearing aid delivery costs were estimated on the basis of program records and an empirical assessment of health personnel time input. Household costs for seeking and undergoing hearing health care were collected with a questionnaire, administered to the parents of the child. Data were collected at three study sites representing primary, secondary and tertiary care levels.

**Results:**

Total screening and hearing aid delivery costs ranged between RMB70,000 (US$9,000) and RMB133,000 (US$17,000) in the three study sites. Health care cost per child fitted ranged from RMB5,900 (US$760) at the primary care level, RMB7,200 (US$940) at the secondary care level, to RMB8,600 (US$1,120) at the tertiary care level. Household costs were only a small fraction of the overall costs. Cost per child fitted ranged between RMB1,608 and RMB2,812 (US$209–US$365), depending on perspective of analysis and study site. The program was always least costly in the primary care setting.

**Conclusion:**

Hearing screening and the delivery of hearing aids in China is least costly in a primary care setting. Important questions remain concerning its implementation.

## Background

The burden of hearing loss among children is large. According to WHO estimates, some 175 million children (up to age 15 years) suffer from mild or greater hearing loss globally, an estimated 61 million children from moderate or greater hearing loss, and 7 million children from severe or profound hearing loss [1]. Hearing loss in childhood may have adverse consequences for optimum childhood development, especially for language acquisition and educational achievement [2]. The provision of hearing aids has been proposed as an effective approach, but a large proportion of all children with hearing disorders go undetected, and as a result relatively few children are fitted with hearing aids [3]. WHO estimates that only one-tenth of the global population in need of hearing aids actually receives them [3].

Active screening for hearing disorders among children has been suggested as an effective strategy to increase case detection, and a number of studies in a range of countries have shown its feasibility in combination with provision of hearing aids [3]. However, no study has systematically evaluated the total costs and the cost per child fitted with hearing aids of such programs. There is an urgent need for research to answer these questions [3].

In response to these observations, this paper reports on the costs of a school-based screening program targeting 216,000 children of age 5–14 years, and the provision of hearing aids to 206 children at three different service levels, in China. This study is part of a number of WWHearing pilot projects on the delivery of hearing aids in developing countries [4].

## Methods

The study evaluated three different programs:

1. Active screening of 71,756 school children in the city of Beijing, and the delivery of hearing aids at the tertiary care level (ie, the China Rehabilitation Research Centre for Deaf Children).

2. Active screening of 88,326 school children in the city of Nanning, southern China, and the delivery of hearing aids at secondary care level (ie, the Nanning Rehabilitation Centre).

3. Active screening of 56,000 school children in the city of Guigang, Guangxi province, southern China, and the delivery of hearing aids at the primary care level (ie, the Guigang Rehabilitation Centre).

In all programs, screening at schools was carried out in two stages. First, questionnaires were sent to the schools to be completed for each child, by the teacher and the parents. Second, on the basis of these questionnaires, children were selected for further screening at schools – by audiologists in programs 1 and 2, and by trained hearing health care workers in program 3. Workers in program 3, all of whom had a background as primary school teachers, attended a three week training program on basic hearing health care, including basic audiometry, ear mould impression taking and hearing aid fitting. Eligible children were then referred to the rehabilitation centers in the respective study sites for further consultation. Eligibility was determined with reference to WHO Guidelines for Hearing Aids and Services for Developing Countries [3], with priority given to children with an average hearing impairment in the better hearing ear in the range 31 to 80 dBHL in the frequency range 500 Hz to 4000 Hz.

At the first visit to the respective rehabilitation centers, children received a physical and a standard audiometric examination. Children were referred in cases where disorders were not treatable with hearing aids. If the child was found to be eligible for hearing aids, an ear mould impression was taken. At a second visit, a digital, programmable hearing aid was fitted. The child was followed up during two visits at five weeks and 3 months post-fitting. In programs 1 and 2, all consultations were carried out by audiologists, and ear moulds were taken and prepared by an ear mould technician. In program 3, the trained hearing health care workers carried out the consultations and took the ear impressions; the impressions were then sent for conversion into ear moulds at Nanning Rehabilitation Centre. Based on the diagnostic audiological assessment, hearing aids were programmed to NAL-NL1 prescription standards [5] and fitted by the audiologists and hearing care workers involved in the project. Evaluation of each individual fitting was later performed and this included aided hearing threshold measures, speech detection testing and child, parent and teacher questionnaires. Results from these measures will be considered in a future research report. The hearing health care service was provided free of health care costs to the children and their families.

The study had a prospective design, in which all children were followed during the course of their inclusion in the program. The costing analysis followed WHO guidelines on costing analysis [6] where possible, and was based on the ingredient approach, ie, separate reporting of prices and quantities. All costs were discounted against a 3% rate, with base year 2006. Health care costs included those of screening and hearing aid delivery, and were estimated on the basis of detailed assessments of program inputs and an empirical assessment of health personnel time input. The latter was obtained through a simple registration of the time that the health personnel (audiologists, hearing worker, or ear mould technician, depending on the study site) spent on care for each child. These data were collected for all programs, but for program 3 only in one site. Household costs for seeking and undergoing care were collected with a questionnaire, administered by the health personnel to the parents of the child. This included questions not only on costs of travel and lodging, but also on foregone income, ie, time lost because of the program. In addition, household treatment seeking patterns prior to the present programs were assessed, including questions on associated costs. These data were collected for all programs.

Guidelines on costing analysis typically advocate adoption of the societal perspective in the calculation of costs and effects [6,7]. This refers to the inclusion of all changes in resource use, no matter who is paying the costs, and includes health care costs (ie, those that accrue to the health care sector) and household costs (ie, those that accrue to patients and families). However, in practice, costing analysis often only includes health care costs, and one of the reasons for this is the difficulty of valuing and, therefore, estimating time costs [6]. As a compromise, we report the cost of the hearing screening program for: i) including health systems costs only; and ii) including both health care and household costs. Household costs refer here only to the costs of *seeking and undergoing care in the context of the present school-based screening program*. In addition, we separately report household costs of seeking and undergoing care before the child was included in the school-based screening program.

## Results

The total number of children screened at schools ranged from 56,000 (Guigang), 71,756 (Beijing) to 88,326 (Nanning). At schools, a proportion of these were examined by audiologists (in Beijing and Nanning) or trained health workers (in Guigang), and of these children, some were referred to a rehabilitation centre for consultation. Subsequently, 52 (Beijing), 76 (Nanning) and 79 (Guigang) children were fitted with hearing aids (Table 1).

**Table 1:** Overview of flow of children at the study sites

	Beijing	Nanning	Guigang	Total
Number of children screened at schools by questionnaires	71,756	88,326	56,000	216,082
Number of children screened at schools by audiologist/health worker	10,853	33,178	314	44,345
Number of children referred to rehabilitation centre for consultation	55	70	54	179
Number of children fitted with hearing aid	37	67	44	148
Number of children fitted per 100,000 screened	52	76	79	68

The health care costs for the school-based screening included fixed and variable costs (Table in additional file 1). Fixed costs are defined here as costs that do not vary with the number of children screened, and include costs of personnel involved in the program (such as that of management, and of trained health workers as employed by the program in Guigang), equipment (such as portable audiometers and otoscopes purchased by the program), and materials (such as printing and distribution of questionnaires). These costs range from RMB24,667 in Beijing, to RMB33,226 in Nanning. Variable costs are defined here as costs that do vary with the number of children screened in schools. Beijing and Nanning carry variable personnel costs (including costs of audiologists for screening, consultation and follow-up activities, ear mould technicians, and hearing technicians), and these are absent in Guigang. Variable equipment costs (such as diagnostics, and the hearing aid itself) and material costs (such as those associated with ear impressions and ear mould manufacture) differ between the sites. Costs of outpatient visits are highest in Beijing, followed by Nanning and Guigang. Total variable costs, per child fitted with hearing aids, ranged from around RMB973 in Guigang, to around RMB1,500 or more in both Beijing and Nanning.

The total health care costs per child fitted with hearing aids is the sum of average fixed costs and (average) variable costs, and ranged from RMB1608 (US$209) in Guigang, RMB1,978 (US$257) in Nanning, to RMB2,361 (US$306) in Beijing. Total costs of the school-based screening program ranged from around RMB70,000 (US$9,000) in Guigang to more than RMB132,000 (US$17,000) in Nanning (Table 2).

**Table 2:** Cost of school-based screening

	Beijing		Nanning		Guigang	
			
**Health care costs**	RMB	US$	RMB	US$	RMB	US$
Cost per child fitted with hearing aid						
Average fixed costs (a)*	667	87	496	64	635	82
Average variable costs (b)**	1,694	220	1,482	192	973	126
Average total health care costs (c = a+b)	2,361	306	1,978	257	1,608	209
Number of children fitted with hearing aid (d)	37		67		44	
Total health care costs (e = c*d)	87,351	11,330	132,530	17.189	70,771	9,179
						
**Household costs**						
Cost per child fitted with hearing aid (f)	451	59	154	20	145	19
Number of children fitted with hearing aid (d)	37		67		44	
Total household costs (g = f*d)	16,703	2,166	10,308	1.337	6,371	826
						
**Cost per child fitted with hearing aids**						
Health care perspective (j = e/d)	2.361	306	1.978	257	1.608	209
Societal perspective (k = (e+g)/d)	2.812	365	2.132	277	1.753	227

* Calculated as (A+C/number of children fitted), with A and C referring to cost categories in Table 2.

** Calculated as (B1+B2+B3), with B1, B2 and B3 referring to cost categories in Table 2.

Household costs related to seeking and undergoing care in the present program are reported in Table 3, and summarized in Table 2. The costs for all four visits ranged from RMB145 in Guigang to RMB451 in Beijing. Overall, transport costs constitute the largest share of these costs, from 38% in Beijing and Nanning, to 48% in Guigang.

**Table 3:** Household costs of seeking and undergoing care in present program

Visit	Beijing		Nanning		Guigang	
			
	N	Mean costs	N	Mean costs	N	Mean costs
First visit						
Registration fees, drugs, tests, hearing aids etc	50	0	67	0	50	0
Family food and lodging costs	50	54	67	44	50	7
Family transport costs	50	54	67	32	50	22
Family income lost because of seeking care	50	29	67	14	50	14
Total costs		137		91		43
Second visit						
Registration fees, drugs, tests, hearing aids etc	31	0	43	0	50	0
Food and lodging costs	31	42	43	5	50	7
Transport costs	31	47	43	22	50	22
Family income lost because of seeking care	31	40	43	11	50	14
Total costs		128		38		42
Third visit						
Registration fees, drugs, tests, hearing aids etc	18	0	2	0	17	0
Food and lodging costs	18	17	2	0	17	2
Transport costs	18	37	2	3	17	13
Family income lost because of seeking care	18	39	2	10	17	15
Total costs	93	13	30			
						
Total costs of all visits*	451	154	145			

* Calculated as (A+C/number of children fitted), with A and C referring to cost categories in Table 2.

** Calculated as (B1+B2+B3), with B1, B2 and B3 referring to cost categories in Table 2.

From the societal perspective – adding up health care costs and household costs – cost per child fitted was between RMB1,753–2,812 (US$227–US$365) in all sites (Table 2). The cost per child fitted was always highest in Beijing, and lowest in Nanning (Table 2).

In addition, we reported household costs of seeking and undergoing care before the child was screened and fitted with a hearing aid in one of the rehabilitation centers. The majority of the children were seen before by other health providers (ranging from 54% in Guigang, to 91% in Nanning, to 100% in Beijing), and among those, most had been seen by traditional healers (Table 4). Very few were seen by governmental doctors. Costs associated with transportation, food and lodging, income lost because of seeking and undergoing care were relatively low.

**Table 4:** Treatment seeking patterns and household costs *prior *to inclusion in the program

**Variable**	Beijing		Nanning		Guigang	
			
**Treatment seeking pattern**	N	%	N	%	N	%
**First place visited elsewhere**						
Traditional healer	47	94	36	54	27	50
Faith healer/religious person	3	6	5	7	0	0
Chemist (e.g. pharmacy)	0	0	12	18	0	0
Village health worker/nurse practitioner	0	0	4	6	0	0
Goverment doctor	0	0	1	1	0	0
Private doctor	0	0	2	3	0	0
Other	0	0	1	1	0	0
No other place	0	0	6	9	23	43
Missing	0	0	0	0	4	7
Total	50	100	67	100	54	100
**Second place visited elsewhere**						
Traditional healer	34	68	30	45	2	4
Faith healer/religious person	4	8	6	9	0	0
Chemist (e.g. pharmacy)	0	0	4	6	0	0
Village health worker/nurse practitioner	0	0	2	3	0	0
Special shop selling hearing aids	0	0	4	6	0	0
No other place	12	24	21	31	48	89
Missing	0	0	0	0	4	7
Total	50	100	67	100	54	100
**Third place visited elsewhere**						
Traditional healer	23	46	17	25	1	2
Faith healer/religious person	1	2	2	3	0	0
Chemist (e.g. pharmacy)	0	0	1	1	0	0
Special shop selling hearing aids	1	2	2	3	0	0
Other	0	0	2	4	0	0
No other place	25	50	43	63	49	91
Missing	0	0	0	0	4	7
Total	50	100	67	100	54	100
						
**Household costs**	N	RMB	N	RMB	N	RMB
**First place visited elsewhere**						
Registration fees, drugs, tests, hearing aids etc.	50	5,852	62	1,259	28	6,054
Family transportation, food and lodging	50	789	62	116	28	358
Family income lost because of seeking care	50	364	61	84	28	187
***Second place visited elsewhere***						
Registration fees, drugs, tests, hearing aids etc.	38	7,936	47	2,380	2	5,007
Family transportation, food and lodging	38	780	47	110	2	508
Family income lost because of seeking care	38	217	47	55	1	16
**Third place visited elsewhere**						
Registration fees, drugs, tests, hearing aids etc.	25	3,862	25	1,465	1	18
Family transportation, food and lodging	25	842	25	61	1	19
Family income lost because of seeking care	25	98	25	38	1	20

## Discussion

Implementation of a school-based screening program for hearing disorders, including the delivery of hearing aids, was, per child fitted, least costly when organized at the primary care level (Guigang). However, differences with implementation of the program at the secondary (Nanning) and tertiary (Beijing) care level were only modest. Five obvious and less evident reasons may explain these differences, and the results should be interpreted in this context.

The first and most obvious reason was that of differences in care level. The rehabilitation centre is Beijing is a tertiary care centre which carries relatively high personnel and building costs compared to primary and secondary care levels, as reflected in the differences in unit costs per outpatient visit. The second reason would seem to be the employment of trained hearing health care workers at the primary care level in Guigang versus the utilization of specialised staff at the secondary or tertiary care level. However, our detailed calculations show that the total personnel costs at secondary and tertiary care level (by multiplying the average personnel cost per child fitted by the number of children fitted) were more or less similar to that of the trained health worker. Thirdly, cost differences may relate to differences in capacity utilization of equipment at the different sites. For example, the rehabilitation centre in Beijing sees more clients on an annual basis, and the average costs per client of equipment such as hearing test rooms and hearing test computers are, therefore, relatively low. Fourthly, cost differences may also reflect artificial differences. For example, distribution costs of questionnaires were much higher in Nanning than in the other two sites, and it is not known whether this reflects technical inefficiencies (ie, waste of resources) that makes the comparison between sites uneven. However, when adapting our analysis for those 'artificial' differences in unit costs, we only found a marginal impact on average cost per child fitted. Finally, cost differences may also be related to the output of the screening program in terms of children fitted with hearing aids, as compared to number of children screened. The number of children fitted with hearing aids was much higher in Guigang and Nanning (75–80/100,000 children screened) than in Beijing (52/100,000). However, when we adapted our analysis for those differences in output, we only found a marginal impact on average cost per child fitted.

A number of additional issues need to be taken into account when interpreting study results. Firstly, if screened school children are referred for consultation, but are not found eligible for hearing aids, the rehabilitation centre in Beijing provides additional diagnostic tests for and treatment of ear-related health problems as a standard procedure. Related costs were not included in the analysis, as the decision to carry out these diagnostic tests and treatment stands by itself [6] and should be subjected to a separate economic analysis. However, related costs may be significant and equally relevant in similar contexts, and policy makers should take these into account when making decisions about the funding of the programs at hand. Secondly, the screening program in this study resulted in fewer detected cases in comparison to previous studies on incidence and prevalence of hearing disorders in school children in both industrialized and developing countries [8,9]. However, questionnaire-based surveys of hearing loss typically have low yield [10,11]. Zhang et al. [12] noted a prevalence rate for hearing disability in young Chinese children of only 0.155% using an initial questionnaire/interview method. Bu, Li & Driscoll [13] found a questionnaire approach gave poor overall accuracy in detecting hearing loss in a group of Chinese school children. In addition, middle ear disorder – a very common cause of mild and moderate hearing loss in Western school children – may not be as prevalent in Chinese school children [14,15]. Many children with severe or profound hearing loss may not attend normal school and, hence, may have been excluded from the screening program. In addition, recall bias is another factor that may affect parent and teacher questionnaire responses. A separate report will assess the validity of the hearing screening questionnaire used in the present program.

This paper evaluated a school based screening program in combination with the provision of hearing aids, and it thereby targeted children of age five years and older. However, hearing loss should ideally be detected as early as possible, preferably in a neonatal screening program, and the present program may be criticized for failing to do so. However, neonatal screening programs are not always easy to implement in developing countries [2]. Also, school-based screening is still needed to identify children who develop hearing loss after the early months of life.

Our study has evaluated the cost per child fitted with hearing aids, and did not assess related health effects. As a consequence, our approach does not differentiate between the capacities of the primary, secondary and tertiary care levels to diagnose hearing problems and fit hearing aids. If tertiary settings provide better care, this may reflect in a higher effectiveness of hearing aids, and the program would, therefore, be rendered more cost-effective. Research has been initiated to document the effectiveness of primary, secondary and tertiary care levels in the same sites as included in the present study. Our results, in terms of cost per child fitted with hearing aids, should therefore be interpreted with caution.

This paper presented cost estimates from both the health care (including only health care costs) and the societal perspective (including health care and household costs). Our results show that household costs were significant (up to RMB415 or US$59 in Beijing) but only a fraction of total costs as estimated from the societal perspective (ranging from 13% in Guigang to 21% in Beijing). The choice of perspective does not change our study conclusions.

Whether the programs can be adapted to render them less costly was not a topic for the present study, but observations of the program process indicate scope. Firstly, most of the resource inputs at the primary care level are fixed (ie, independent of the number of children screened and fitted), and this means that economies of scale can be obtained by increasing the catchment areas of the rehabilitation centers. Secondly, questionnaires were initially distributed to school children as part of the screening process, but study sites abandoned the use of these questionnaires during the project because of concerns regarding their validity.

An interesting observation is that almost all households had visited a different health provider before being included in the program under study. This indicates that the hearing disorders of the children in the present program did not go undetected prior to the school-based screening program, but rather that parents did not seek care with modern health providers. One possible explanation is that hearing aids were *not *delivered free of charge prior to the present program (as they *were *in the present program), and were not affordable to households. This poses a question concerning the relevance of the screening program, and whether the delivery of (highly) subsidized hearing aids, in combination with an (school-based) information campaign publicizing their availability, may not reach the same objective.

Obviously, the nature of the screening program – including all 5–14 year old children – does not require annual repetition in the same population. Policy makers, therefore, need to decide on whether the program should be repeated every, eg, 10 years, or should annually target the youngest age cohorts only, ie, new 5 year old cohorts. More detailed costing and cost-effectiveness analysis can be implemented to support these decisions.

## Conclusion

In conclusion, screening and delivery of hearing aids to school children in China is, per child fitted, least costly in a primary care setting. Important questions remain regarding its implementation.

## Competing interests

The authors declare that they have no competing interests.

## Authors' contributions

RB, LJ and SXB designed the study. WLD, GXH, TBY, HR, WXL collected the data. RB analysed the data. All authors contributed to the drafting of the manuscript.

## Pre-publication history

The pre-publication history for this paper can be accessed here:



## Supplementary Material

Additional file 1 **Supplementary table**. Resource utilization, unit costs and total costs of school screening program and fitting of hearing aids. Click here for file
