# Supplementary material for: Costs of screening children for hearing disorders and delivery of hearing aids in China
Source: BMC Health Serv Res. 2009 Apr 16;9:64. doi: 10.1186/1472-6963-9-64 (PMC2679736; doi:10.1186/1472-6963-9-64)
Supplement: Additional file 1 — Supplementary table. Resource utilization, unit costs and total costs of school screening program and fitting of hearing aids. [file 1472-6963-9-64-S1.doc]

**Table**. Resource utilization, unit costs and total costs of school screening program and fitting of hearing aids

|  |  |  | Beijing |  |  |  | Nanning |  |  |  | Guigang |  |  |
| --- | --- | --- | --- | --- | --- | --- | --- | --- | --- | --- | --- | --- | --- |
| A. Fixed costs | |  | Quantity* | Unit price** | Total costs |  | Quantity* | Unit price** | Total costs |  | Quantity* | Unit price** | Total costs |
|  |  |  |  |  |  |  |  |  |  |  |  |  |  |
|  | Personnel | Director (days) | 10 | 80,000 | 3,333 |  | 10 | 40,000 | 1,667 |  | NA |  |  |
|  |  | Health worker (days) | NA |  |  |  | NA |  |  |  | 240 | 12,000 | 12,000 |
|  |  | Total |  |  | 3,333 |  |  |  | 1,667 |  |  |  | 12,000 |
|  |  |  |  |  |  |  |  |  |  |  |  |  |  |
|  | Equipment | Portable audiometer | 6 | 1,310 | 7,861 |  | 5 | 1,310 | 6,551 |  | NA |  |  |
|  |  | Otoscope | 1 | 193 | 193 |  | 5 | 82 | 410 |  | 1 | 82 | 82 |
|  |  | Hearing test room | NA |  |  |  | NA |  |  |  | 1 | 4.367 | 4,367 |
|  |  | Total |  |  | 8,053 |  |  |  | 6,961 |  |  |  | 4,449 |
|  |  |  |  |  |  |  |  |  |  |  |  |  |  |
|  | Material | Printing questionnaire | NA | NA | 5,000 |  | NA | NA | 5,000 |  | NA | NA | 3,750 |
|  |  | Distribution of questionnaire | NA | NA | 8,300 |  | NA | NA | 19,600 |  | NA | NA | 7,750 |
|  |  | Total |  |  | 13,300 |  |  |  | 24,600 |  |  |  | 11,500 |
|  | Total |  |  |  | 24,687 |  |  |  | 33,228 |  |  |  | 27,949 |
|  |  |  |  |  |  |  |  |  |  |  |  |  |  |
| B. Variable costs | |  |  |  |  |  |  |  |  |  |  |  |  |
|  | B1. Personnel | Audiologist for screening at school (days)**** | 28 | 167 | 91 |  | 28 | 167 | 62 |  | NA |  |  |
|  |  | Audiologist (1st visit) | 1.47 | 40,000 | 30.58 |  | 1.06 | 17,500 | 9.66 |  | NA |  |  |
|  |  | Audiologist (2nd visit) | 1.10 | 40,000 | 22.83 |  | 0.70 | 17,500 | 6.38 |  | NA |  |  |
|  |  | Audiologist (3rd visit) | 0.88 | 40,000 | 18.41 |  | 1.00 | 17,500 | 9.11 |  | NA |  |  |
|  |  | Audiologist (4th visit) | 0.88 | 40,000 | 18.41 |  | 1.00 | 17,500 | 9.11 |  | NA |  |  |
|  |  | Ear mould technician (1st visit, taking mould) | 0.84 | 30,000 | 13.07 |  | 0.73 | 15,000 | 5.70 |  | NA |  |  |
|  |  | Ear mould technician (making mould) | 1.00 | 30,000 | 15.63 |  | 2.27 | 15,000 | 17.73 |  |  |  |  |
|  |  | Ear mould technician (2nd visit, fitting mould) | 0.16 | 30,000 | 2.52 |  | 0.15 | 15,000 | 1.17 |  | NA |  |  |
|  |  | Ear mould technician (3rd visit) | 0.08 | 30,000 | 1.30 |  | 0.00 | 15,000 | 0.00 |  | NA |  |  |
|  |  | Ear mould technician (4th visit) | 0.08 | 30,000 | 1.30 |  | 0.00 | 15,000 | 0.00 |  | NA |  |  |
|  |  | Hearing test technician (1st visit) | 0.54 | 30,000 | 8.42 |  | 0.73 | 15,000 | 5.70 |  | NA |  |  |
|  |  | Hearing test technician (2nd visit) | 0.46 | 30,000 | 7.13 |  | 0.48 | 15,000 | 3.75 |  | NA |  |  |
|  |  | Hearing test technician (3rd visit) | 0.47 | 30,000 | 7.31 |  | 0.50 | 15,000 | 3.91 |  | NA |  |  |
|  |  | Hearing test technician (4th visit) | 0.47 | 30,000 | 7.31 |  | 0.50 | 15,000 | 3.91 |  | NA |  |  |
|  |  | Total |  |  | 245 |  |  |  | 138 |  |  |  | NA |

**Table**. Resource utilization, unit costs and total costs of school screening program and fitting of hearing aids (continued)

|  |  |  | Beijing |  |  |  | Nanning |  |  |  | Guigang |  |  |
| --- | --- | --- | --- | --- | --- | --- | --- | --- | --- | --- | --- | --- | --- |
| B. Variable costs | |  |  |  |  |  |  |  |  |  |  |  |  |
|  |  |  | Quantity* | Unit price** | Total costs |  | Quantity* | Unit price** | Total costs |  | Quantity* | Unit price** | Total costs |
|  | B2. Equipment | Pure tone audiometer (per patient screened) | 1 | 10.25 | 10.25 |  | 1 | 40.18 | 40.18 |  | 1 | 50.22 | 50.22 |
|  |  | Otoscope | NA*** |  |  |  | NA*** |  |  |  | NA*** |  |  |
|  |  | DPOAE acoustic | 1 | 99.26 | 99.26 |  | 1 | 99.26 | 99.26 |  | NA |  |  |
|  |  | Acoustic Immitance measurement | 1 | 20.94 | 20.94 |  | 1 | 20.94 | 20.94 |  | NA |  |  |
|  |  | Synthetic treatment table | 1 | 3.80 | 3.80 |  | 1 | 3.80 | 3.80 |  | NA |  |  |
|  |  | Equipment for taking mould | 1 | 6.00 | 6.00 |  | 1 | 6.00 | 6.00 |  | NA |  |  |
|  |  | Equipment for making mould | 1 | 39.61 | 39.61 |  | 1 | 102.88 | 102.88 |  | NA |  |  |
|  |  | Hearing aid | 1 | 585.96 | 585.96 |  | 1 | 585.96 | 585.96 |  | 1 | 586.96 | 585.96 |
|  |  | Computer and Hi-Pro software | 1 | 3.34 | 3.34 |  | 1 | 14.29 | 14.29 |  | 1 | 14.29 | 14.29 |
|  |  | Hearing test room | 2 | 1.46 | 2.91 |  | 2 | 21.84 | 43.67 |  | NA |  |  |
|  |  | Total |  |  | 772.07 |  |  |  | 916.97 |  |  |  | 650.47 |
|  |  |  |  |  |  |  |  |  |  |  |  |  |  |
|  | B3. Materials | Materials for taking mould impression | 1 | 0.08 | 0.08 |  | 1 | 0.08 | 0.08 |  | NA |  |  |
|  |  | Materials for making mould | 1 | 35.00 | 35.00 |  | 1 | 50.00 | 50.00 |  | NA |  |  |
|  |  | Battery provision | 1 | 1.00 | 1.00 |  | 1 | 1.00 | 1.00 |  | 1 | 1.00 | 1.00 |
|  |  | Battery provision at 3 months | 1 | 1.00 | 1.00 |  | 1 | 1.00 | 1.00 |  | 1 | 1.00 | 1.00 |
|  |  | Total |  |  | 37.08 |  |  |  | 52.08 |  |  |  | 2.00 |
|  |  |  |  |  |  |  |  |  |  |  |  |  |  |
|  | B4. Outpatient visits | 1st visit | 1.47 | 147.69 | 216.81 |  | 1 | 99.84 | 105.83 |  | 0.50 | 70.38 | 35.19 |
|  |  | 2nd visit | 1.10 | 147.69 | 161.84 |  | 1 | 99.84 | 69.89 |  | 0.80 | 70.38 | 56.30 |
|  |  | 3rd visit | 0.88 | 147.69 | 130.54 |  | 1 | 99.84 | 99.84 |  | 0.50 | 70.38 | 35.19 |
|  |  | 4th visit | 0.88 | 147.69 | 130.54 |  | 1 | 99.84 | 99.84 |  | 0.50 | 70.38 | 35.19 |
|  |  | Total |  |  | 640 |  |  |  | 375 |  |  |  | 162 |
|  |  |  |  |  |  |  |  |  |  |  |  |  |  |
|  | B5. Other | Contracting out ear mould making in Nanning |  |  |  |  |  |  |  |  | 1.00 | 159 | 158.88 |
|  |  |  |  |  |  |  |  |  |  |  |  |  |  |
|  | Total |  |  |  | 1,694 |  |  |  | 1,482 |  |  |  | 973 |
| * Personnel time and time of outpatient visits in hours | | |  |  |  |  |  |  |  |  |  |  |  |
| ** Personnel salaries per year; costs per outpatient visit per hour | | |  |  |  |  |  |  |  |  |  |  |  |
| *** Included as fixed costs | | |  |  |  |  |  |  |  |  |  |  |  |
| **** Variable cost per child fitted | | |  |  |  |  |  |  |  |  |  |  |  |
